# Oral magnesium supplements for cancer treatment‐induced hypomagnesemia: Results from a pilot randomized trial

**DOI:** 10.1002/hsr2.443

**Published:** 2021-12-14

**Authors:** Arif Awan, Bassam Basulaiman, Carol Stober, Mark Clemons, Dean Fergusson, John Hilton, Waleed Al Ghareeb, Rachel Goodwin, Mohammed Ibrahim, Brian Hutton, Lisa Vandermeer, Ranjeeta Mallick, Michael M. Vickers

**Affiliations:** ^1^ Department of Medicine, Division of Medical Oncology The Ottawa Hospital Cancer Centre Ottawa Ontario Canada; ^2^ Cancer Therapeutics Program Ottawa Hospital Research Institute Ottawa Ontario Canada; ^3^ Department of Medicine University of Ottawa Ottawa Ontario Canada; ^4^ Clinical Epidemiology Program Ottawa Hospital Research Institute Ottawa Ontario Canada

**Keywords:** chemotherapy, EGFR inhibitors, hypomagnesemia, integrated consent model

## Abstract

**Background and Aims:**

Optimal management of cancer treatment‐induced hypomagnesemia (hMg) is not known. We assessed the feasibility of using a novel pragmatic clinical trials model to compare two commonly used oral Mg replacement strategies.

**Methods:**

Patients with grade 1 to 3 hMg while receiving either platinum‐based chemotherapy or epidermal growth factor receptor inhibitors (EGFRI) were randomized to oral magnesium oxide (MgOx) or oral magnesium citrate (MgCit). The trial methodology utilized the integrated consent model. Feasibility would be successful if; accrual rate was ≥5 patients a month and if measures of patient and physician engagement, were > 50%. Secondary endpoints included; comparison of Mg levels, cardiac arrhythmias, and rates of treatment delay/hospitalizations.

**Results:**

From July 2016 to December 2017, an average of 1 patient a month was accrued. All 15 eligible and approached patients consented to participate in the study (100% engagement) and 7/15 were randomized to MgOx and 8/15 to MgCit. The percentage of physicians who approached patients for the study was 4 of 6 (66.6% engagement). The mean slope of change in Mg (mmol/L/day) was 0.0022 (95% CI: −0.0001 to 0.0044) for MgOx and 0.0006 (95% CI, −0.0012 to 0.0024) for MgCit (*P* = .2123). Three patients (20%) required IV magnesium while on the study (2 MgCit and 1 MgOx). Grade 1 diarrhea occurred in 3 patients in the MgCit arm.

**Conclusion:**

Despite oral magnesium tolerability and meeting most of its feasibility endpoints, this study did not meet its target accrual rate. Alternative designs would be necessary for a definitive efficacy study.

## 1 INTRODUCTION

Hypomagnesemia (hMg) is a common side effect of both platinum‐containing chemotherapy and epidermal growth factor receptor inhibitors (EGFRIs). The reported incidence is approximately 90% in patients receiving cisplatin, 27% with panitumumab (pmab), and 18% with cetuximab (cmab).^1^, ^2^ The consequences of hMg include fatigue, nausea and vomiting, neuromuscular changes, mental status changes and cardiac arrhythmias, potentially resulting in treatment delays, and compromised treatment efficacy.^3^

Despite the high incidence of hMg, little is known regarding effective management.^4^, ^5^, ^6^ In most patients with severe (grade 3/4) hMg, high‐dose intravenous (IV) magnesium replacement is commonly used, however, this does not achieve sustainable magnesium repletion beyond 72 hours, suggesting that such a strategy is both suboptimal and inconvenient for patients.^7^ A recent study of ovarian cancer patients receiving carboplatin showed that in spite of most patients receiving prophylactic IV Mg, there was a high frequency of hMg and future prospective trials were suggested.^8^ Studies of IV Mg in patients with EGFRI‐induced hMg have shown IV Mg may not be beneficial for any grade of hMg and that, despite its use, 97% of patients will have a decline in their magnesium levels over time.^9^ An alternative Mg replacement strategy involves oral supplementation. A recent review of magnesium supplements has suggested that magnesium citrate (MgCit) may have the best bioavailability.^10^ Given this uncertainty, it is not surprising that a survey of oncologists showed that a variety of replacement strategies are used in practice. The majority of respondents used a combination of oral and IV supplements depending on the grade of hMg, with magnesium oxide (MgOx), magnesium rougier, and MgCit being the most commonly used oral agents.^11^

Given the absence of comparative randomized trials assessing the most effective oral supplementation for EGFR and platinum‐induced hMg, the demonstration of clinical equipoise from surveys, the lack of efficacy of high‐dose IV magnesium replacement, and the variable kinetics of different oral replacement strategies, there is a need for robust trials. Unfortunately, performing such trials using traditional clinical trial methodologies are challenging, expensive and unlikely to occur. Our team has been evaluating trial models for comparison of standard of care interventions that are more pragmatic, inexpensive, and practical.^12^ In the current study, we assessed the feasibility of performing a pragmatic clinical trial using this novel methodology for comparing two commonly used oral Mg replacement strategies. In addition, given the continued renal losses of magnesium with ongoing both platinum and EGFRI therapies, we wished to evaluate if oral supplementation would blunt the decline in magnesium levels.^2^, ^9^, ^13^

## 2 METHODS

### 2.1 Study population

Adult patients receiving palliative cisplatin, carboplatin, panitumumab, or cetuximab at the Ottawa Hospital Cancer Centre (Ottawa, Canada) who developed grade 1 to 3 hMg were potentially eligible for this study. Patients were recruited from outpatient clinics and the chemotherapy treatment unit from July 13, 2016 to December 31, 2017 and gave consent to participate using the integrated verbal consent model. Grading of hMg was as per Common Terminology Criteria for Adverse Events (CTCAE) v4.03 with grade 1 defined as < Lower Limit Normal (LLN) to 0.5 mmol/L (1.2 mg/dL), grade 2 as <0.5 to 0.4 mmol/L (<1.2‐0.9 mg/dL), grade 3 as <0.4‐0.3 mmol/L (<0.9‐0.7 mg/dL) and grade 4 as <0.3 mmol/L (<0.7 mg/dL).^14^ Other inclusion criteria included: expectation of receiving ≥2 months of further therapy, potassium within normal limits, ECOG ≤2, ability to swallow tablets/capsules, and ability to provide verbal consent. Exclusion criteria included: grade 4 hMg (<0.3 mmol/L), baseline creatinine >1.5× upper limit of normal (ULN) and current use of oral or IV magnesium supplementation. Patients who received 1 g of magnesium with their standard Cisplatin/Carboplatin chemotherapy regimens were eligible. Pre‐treatment evaluations included standard of care biochemistry (including K^+^, Mg^2+^, creatinine) and an ECG.

### 2.2 Ethical statement

The study was approved by the Ottawa Health Science Network Research Ethics Board (OHSN‐REB) at the Ottawa Hospital. The trial was registered on clinicaltrials.gov (NCT02690012). All procedures performed in studies involving human participants were in accordance with the ethical standards of the institutional research committee and with the 1964 Helsinki declaration and its later amendments or comparable ethical standards.

### 2.3 The ReThinking Clinical Trials (REaCT) program

The development of the REaCT program for comparing standard of care interventions is outlined elsewhere.^12^ Briefly, after exploring the multiple processes and barriers to performing clinical trials, several issues with conventional clinical trials were identified that, if streamlined, could allow for more efficient, and effective pragmatic trials to be considered. The key components considered in the current study included: selection of a clinically relevant and practical question; demonstration of clinical equipoise through surveys of knowledge users and completion of systematic reviews; simply defined study endpoints and use of an integrated consent model (ICM) incorporating oral consent; establishing web‐based randomization and real‐time electronic data capture and management.^12^, ^15^, ^16^, ^17^, ^18^ While this methodology has been used before to compare supportive care and palliative agents, this was the first study to evaluate whether such a methodology was feasible for a real world of an intervention for patients with hMg.

### 2.4 Trial design

This study was a prospective single center, open‐label, randomized (1:1) feasibility pilot trial.

### 2.5 Consent process

Potentially eligible patients were informed about the risks of hMg and the two different standard of care oral Mg replacement strategies available to them. The physician would provide the patient a consent template that briefly outlined the study and explain both the idea of randomization and the patient's right to decline study entry (see Data S1). Patients consented orally and this clinical interaction was documented in the patient's electronic health record.^12^ Informed oral consent was obtained from all individual participants included in the study.

### 2.6 Randomization

Eligible and consenting patients were randomized to either MgOx or MgCit using a web‐based randomization system with permuted block sizes of 2 & 4 developed by the Ottawa Methods Centre. In keeping with real world practice, the dose prescribed depended on the grade of hMg and could be adjusted if the Mg level changed during study participation. For Grade 1 hMg, MgCit 150 mg BID or MgOx 420 mg BID were prescribed, Grade 2 MgCit 300 mg BID or MgOx 420 mg TID and grade 3 either at baseline or during the study, then MgCit 300 mg TID and MgOx 820 mg BID with IV MgSO4 5 g over 3 hours within a week. Patients that developed grade 4 hMg (<0.3 mmol/L) discontinued the study protocol.

### 2.7 Data collection

Data were collected both from the patient's electronic medical record (EMR) and emails sent to the treating physician when the patient returned to the clinic. Mg levels were collected retrospectively every 2 weeks for patients receiving panitumumab/cetuximab and every 3 weeks for those receiving cisplatin/carboplatin.

### 2.8 Primary outcomes

A combination of endpoints was collected to evaluate the feasibility of performing a study with this novel methodology. These included: accrual rates (defined as the percentage of eligible and approached patients who consented to participate in the study) and measures of patient and physician engagement. Patient engagement was defined as the percentage of patients approached for the study that agreed to be randomized to the study intervention, while physician engagement was defined as the percentage of medical oncologists who agreed to participate in the study at study commencement and who approached patients and/or allowed their patients on the study. This study would be deemed feasible if ≥5 patients per month were accrued and the patient and physician engagement was >50%.

### 2.9 Secondary outcomes

Secondary outcomes were clinical in nature and included: comparison of the slope of change in Mg levels (from baseline) over time between the two regimens. In addition, the association of baseline QTc and grade of hypomagnesemia at baseline was assessed as well as the rates of treatment delays and hospital admissions due to hMg.

### 2.10 Exploratory outcomes

A comparison of the proportion of patients in the MgOx vs MgCit groups who received IV magnesium as well as rates of grade 3/4 hypomagnesemia at beginning of each cycle, and a comparison of grade of diarrhea (using NCI CTCAE version 4.03) by treatment arm.

### 2.11 Sample size and statistical analysis

A convenience sample size was calculated from the estimated incidence of hMg at our center. Approximately 242 patients a year receive palliative systemic therapy platinum or EGFR inhibitor‐based at our center. Of these patients around 80% will receive platinum‐based therapy and given the incidence of hMg in this population^1^, ^2^ it was estimated the 10 patients/month would develop hMg. As not all patients would choose to enter the study and others would be ineligible (eg, high creatinine, low potassium) a practical accrual rate of 5 patients per month (ie, 60 patients over a year) was established as being an accrual rate that may allow future expansion of the study with adequate power to compare Mg‐replacement strategies and therefore current analyses were considered exploratory. Study results are presented descriptively, and following the recommendation of the CONSORT extension statement for randomized pilot and feasibility trials.^19^ Patients were stratified by EGFRI or platinum containing systemic therapy. For each participant, a linear regression was fitted to change in Mg level from baseline with time from baseline being the independent variable. The slopes obtained from the regression were compared between groups using Wilcoxon rank sum test. An exploratory sensitivity analysis (mixed effects model) was also performed. All analyses used SAS 9.4 by SAS Institute Inc. Cary, NC, USA.

## 3 RESULTS

**FIGURE 1:**
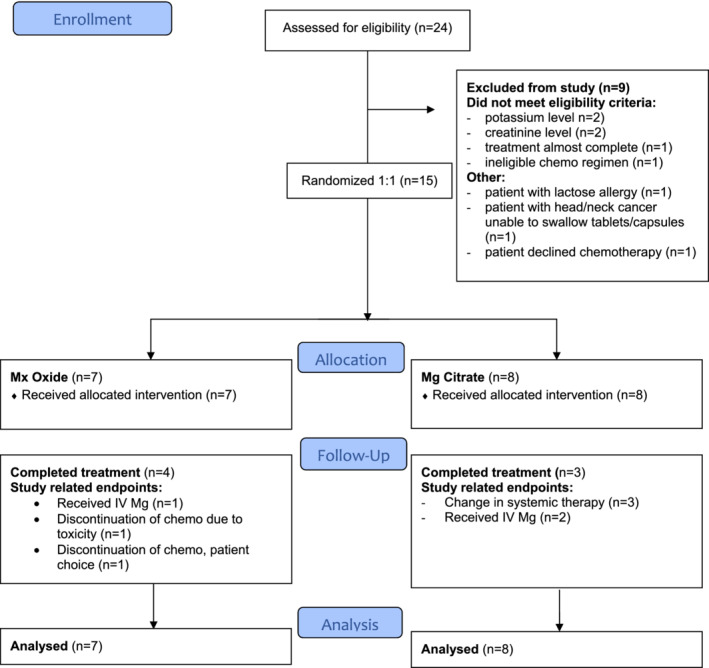
CONSORT flow diagram

The trial ran from July 2016 to December 2017 and during the study period, the trial investigators were contacted about 24 potentially eligible patients. Of these patients, 15 (62.5%) were eligible (Figure 1).

The reasons for ineligibility were; low potassium level,^2^ high creatinine level,^2^ lactose allergy,^1^ inability to swallow tablets/capsules,^1^ patient on the last cycle of planned treatment,^1^ patient declined systemic therapy^1^ and patient not receiving any of the specified therapies.^1^ The baseline characteristics of the study population are shown in Table 1.

**TABLE 1:** Baseline characteristics

	Total	Mg‐citrate	Mg‐oxide
N	15	8 (53.3%)	7 (46.7%)
Median Age (range)	62 (35–85)	67 (35‐85)	62 (39‐72)
Sex			
Male	10	5 (50%)	5 (50%)
Female	5	3 (60%)	2 (40%)
Type of cancer			
Lung	7 (46.7%)	2 (13.3%)	5 (33.3%)
Gastrointestinal	6 (40%)	5 (33.3%)	1 (6.7%)
Head and Neck	2 (13.3%)	1 (6.7%)	1 (6.7%)
Genito‐urinary	0	0	0
EGFRI treatment			
Cetuximab (n, %)	3 (20%)	2 (13.3%)	1 (6.7%)
Panitumumab (n, %)	4 (26.7%)	3 (20%)	1 (6.7%)
Chemotherapy			
Cisplatin (n, %)	4 (26.7%)	2 (13.3%)	2 (13.3%)
Carboplatin (n, %)	4 (26.7%)	1 (6.67%)	3 (20%)
Baseline Mg^2+^ level median, (range)	0.557 (0.44‐0.72) mmol/L	0.59 (0.44‐0.62) mmol/L	0.55 (0.48‐0.72) mmol/L
Grade 1	11 (73.3%)	6 (40%)	5 (33.3%)
Grade 2	4 (26.7%)	2 (13.3%)	2 (13.3%)
Grade 3	0	0	0
QTcF (range)	437.3 (404‐465)	435 (404‐465)	451 (416‐460)

Median age was 62 years (range 35‐85 years) and the percentage of patients randomized to the MgCit and MgOx arms were 53.3% (8/15) and 46.7% (7/15), respectively. The most common tumor types were; lung (7/15, 46.7%), colorectal (6/15, 40%), and Head and Neck (2/15, 13.3%) cancers. The most common systemic treatments at the time of randomization were; cisplatin (4/15, 26.7%), carboplatin (4/15, 26.7%), cetuximab (3/15, 20%), and panitumumab (4/15, 26.7%). The median baseline Mg values and grade of hMg were similar between patients that received MgOx and MgCit.

### 3.1 Primary outcome measures

#### 3.1.1 Actual accrual

Over the study period of 15 months, 15 patients were eligible and entered the study. This was an average of 1 patient a month. Strategies were established to try and increase accrual such as; regular reminder emails to all physicians, posting information about the trial in all chemotherapy treatment units (CTU) so nursing teams were aware, and asking clerical staff to screen all Mg results before patients came to the chemotherapy unit. Following continued discussions with the research team comparing actual with planned accrual as well as diminished funding and the trial being open for over 12 months, the pragmatic decision was made to close the trial after 15 patients were enrolled. Physicians were informally surveyed as to reasons for insufficient accrual. Many noted that the pre‐treatment clinical encounters occurred prior to knowledge of Mg results were available and that they were not often contacted by CTU staff if their patient had hMg. In addition, some felt it was easier to prescribe IV Mg than to engage patients in a clinical trial of oral Mg supplementation.

#### 3.1.2 Patient engagement

Of the 15 potentially eligible and approached patients, all 15 (100%) consented to participate in the study and agreed to the study intervention (100% engagement).

#### 3.1.3 Physician engagement

While all medical oncologists agreed to allow their eligible patients to be approached and participate in the study, the percentage of physicians who actually approached patients was 4 of 6 (66.6% engagement).

#### 3.1.4 Secondary outcomes measures

Clinical endpoint data are reported in Table 2.

**TABLE 2:** Clinical endpoint data

	Overall	Magnesium citrate (n = 8)	Magnesium oxide (n = 7)
Number of treatment delays due to hMg	0	0	0
Hospitalisations related to hMg	0	0	0
Incidence of diarrhea (n) any grade while on trial:	3	3	0
Grade 1			
Grade 2			
Grade 3			
Number of patients receiving IV Mg (not part of the systemic therapy)	3	2	1
Incidence of Grade 4 hypomagnesemia (Mg < 0.3)	0	0	0

#### 3.1.5 Change in magnesium from baseline (Figure 2)

**FIGURE 2:**
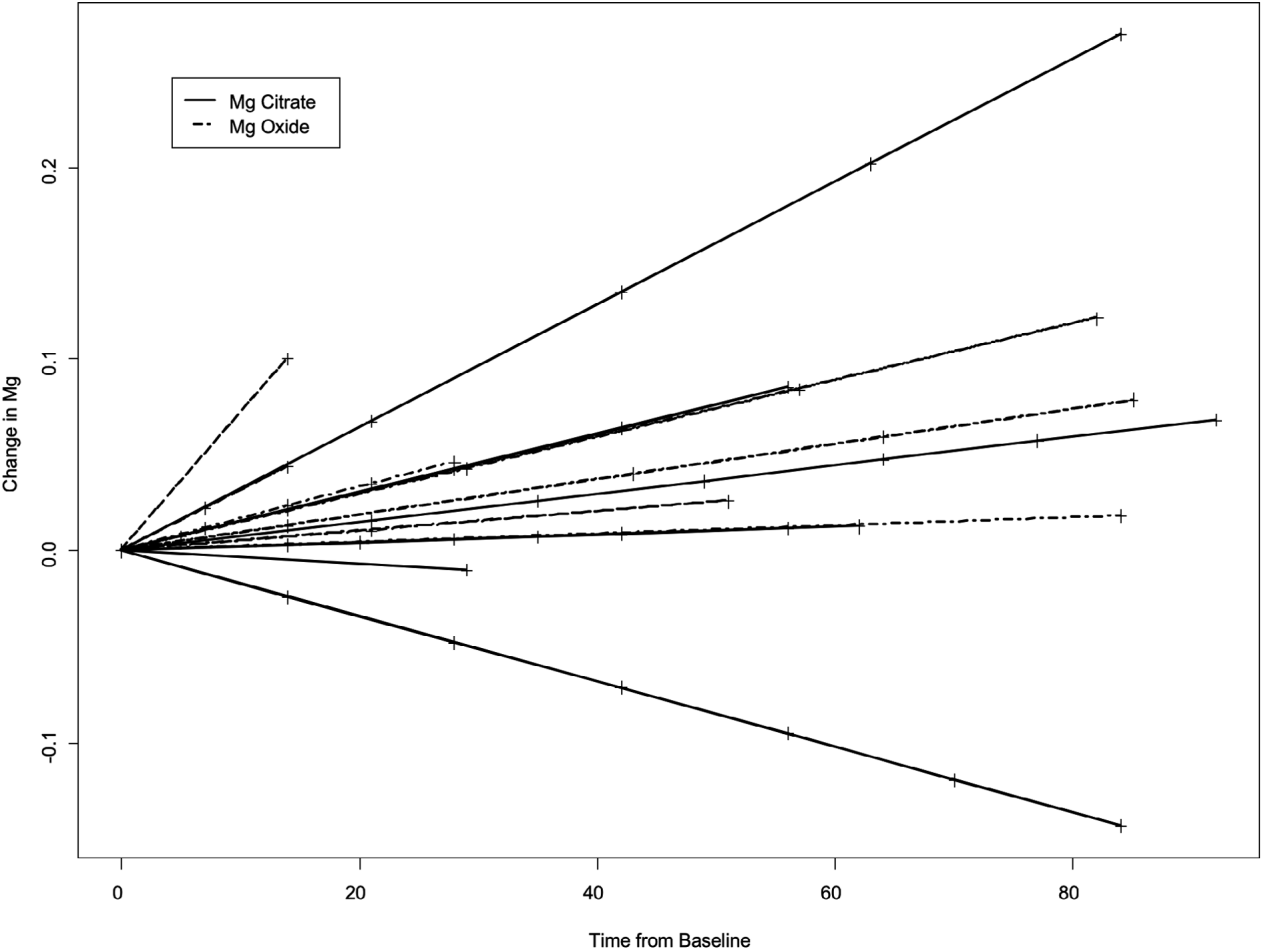
Changes in serum magnesium concentrations from baseline over time (days) during Platinum‐based chemotherapy and EGFRI treatment

**FIGURE 3:**
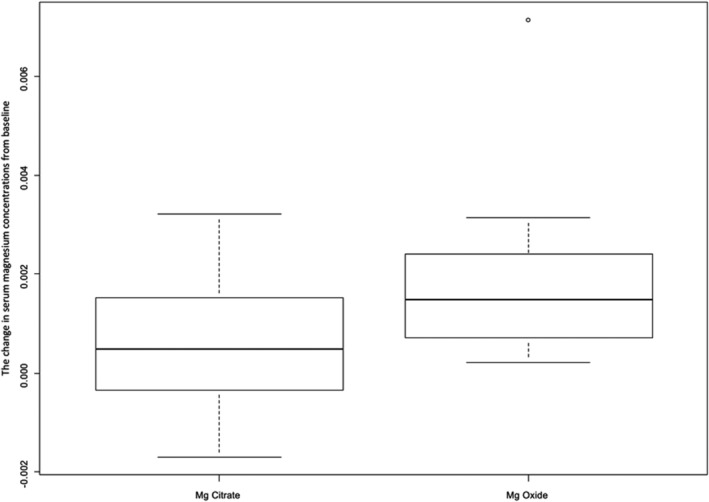
Comparison of the slope of the change in serum magnesium concentrations from baseline in patients receiving magnesium oxide and magnesium citrate

The median (IQR) slope of change in Mg (mmol/day) was 0.0015 (0.0005, 0.0031) for MgOx and 0.0005 (−0.0003, 0.0015) for MgCit (*P* = .2246) (Figure 3).

**FIGURE 4:**
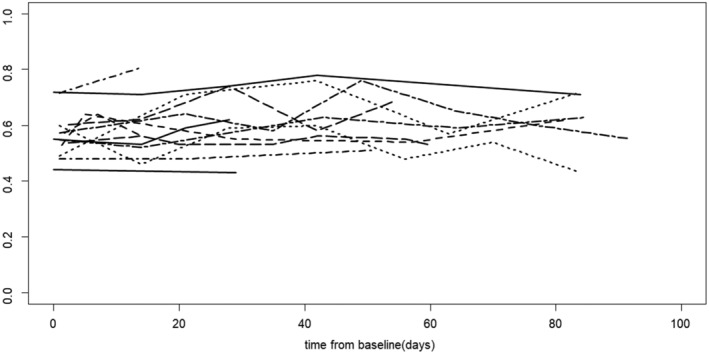
Change in Mg levels for individual patients

The mean difference (95% CI) in slope is −0.0016 (0.002, 0.0012). The sensitivity analysis (mixed effects model) showed similar results. The individual patient plot for change in Mg is shown in Figure 4. There was no Grade 4 hMg during the study.

### 3.2 QTc assessments

Baseline ECGs showed incidental long QTc in 3/15 (20%) patients and with no patients developing arrhythmias on the trial. There was a non‐significant negative correlation between baseline Mg levels and QTc (Frederica) −0.11 (*P* = .63). Follow‐up ECGs were not routinely obtained for a significant number of patients for comparison of on trial effects.

### 3.3 Treatment delays and hMg‐induced hospitalization

There were no treatment delays or hospitalizations for hMg during the study in either study arm.

### 3.4 Exploratory outcomes

#### 3.4.1 Diarrhea

There were 37.5% (3/8) who developed grade 1 diarrhea on MgCit compared to no grade ≥1 diarrhea with MgOx. No patient developed grade 3/4 diarrhea.

#### 3.4.2 Requirements for IV Mg

There were 25% (2/8) patients on MgCit requiring IV magnesium supplementation compared to 14% (1/7) for MgOx (*P* = .1). These IV supplements were received outside of study protocol at the discretion of the treating physicians. No patient developed grade 3/4 hMg prior to the start of each systemic therapy cycle.

## 4 DISCUSSION

The optimal management of treatment induced hMg is unknown.^4^, ^5^, ^6^ This pragmatic trial attempted to determine if the use of an integrated consent model would be feasible to compare two standard of care oral magnesium supplementation strategies.

While the study met its endpoints in terms of physician engagement and patient/physician compliance with the randomized arm, the major challenge was the slow rate of patients being approached for the study. Strategies were used to try to increase study awareness including multiple email reminders to nursing, pharmacy, and medical oncologists. Despite this, the overall number of patients accrued to the study was much lower than expected. The biggest perceived obstacle to identifying and approaching patients for this trial was logistical due to not knowing pre‐treatment magnesium levels during the oncologists clinic visit. Our center does not offer same day chemotherapy and therefore pre‐treatment bloodwork is usually obtained after the clinic visit and within 48 hours of the next systemic therapy. As this was expected, a qualified investigator was available each day to be on‐call to the chemotherapy treatment unit if a patient was identified as having hMg. It is possible that treating oncologists perceived this as too onerous and simply administered IV Mg to their patients as opposed to contacting the on‐call trial team.^16^, ^20^ Another possible strategy could have been to have a research staff screen all chemotherapy patients for eligibility each day. Although this may have led to better accrual, it was contrary to our pragmatic design. Recently, the MAGNET trial reported that prophylactic strategy (as opposed to reactive) of oral Mg gluconate reduced Mg wasting when administered to patients with advanced colorectal cancer treated with EGFRIs.^21^ This trial was successful in randomizing 171 patients and therefore may be a more feasible setting compared to a reactive randomization as was the case in our study.

Although small in size, our trial has important implications for clinical practice. An oral replacement strategy was effective in avoiding IV Mg supplementation since only 3/15 patients subsequently received IV Mg and no patient developed grade 3/4 hMg. This is in keeping with the known renal effects of EGFRIs and MG wasting and the suggestion that saturating the gut with MG may be the optimal management strategy.^22^ Avoidance of IV MG using an oral Mg replacement strategy is also important as it may shorten treatment times for patients and increase efficiency in chemotherapy treatment units. An often‐cited concern with oral magnesium supplementation is the side effect of diarrhea. Notably, we found oral magnesium supplementation to be well tolerated and diarrhea, if experienced, was mild. Further, prior studies with EGFRI have shown a mean decrease of −0.00157 mmol/L/day (95% CI −0.00191 to −0.00123) in magnesium with the negative slope and confidence interval indicating that EGFRI treatments results in reduction of magnesium.^9^ In our study, the slope of magnesium levels increased over time which is also reassuring for clinicians considering an oral replacement strategy. The goal of oral MG replacement should be to prevent further decline in MG as opposed to significant increases in MG levels. A recent retrospective study from Japan also suggests initiation of early MG supplementation if baseline levels are low prior to EGFRI therapy.^23^

While it is recognized that severe hypomagnesemia has been associated with cardiac arrhythmia,^6^ a knowledge user survey of 40 Canadian gastrointestinal medical oncologists regarding EGFRI‐induced hMg management showed that 97.5% of respondents do not assess electrocardiograms (ECGs) in patients who develop hMg despite the potential for serious cardiac complications.^11^ In the current study, 3 (20%) patients (all male) had a baseline QTcF of >450 ms (456‐552 ms). This suggests that patients undergoing therapy with platinums or EGFRIs should have ECGs at baseline and followed closely if abnormal. Unfortunately, few patients had follow‐up ECGs as per protocol, thus limiting further evaluation of change in QTc values over time with changes in magnesium levels. No patient had a cardiac complication and no patient was hospitalized due to complications of hMg. Practicing oncologists should also consider routine calcium measurements since hMg can cause hypocalcemia through impaired PTH release and function.^24^

The limitations of the current study are well recognized and include the open label design, the small sample size, and enrollment at a single cancer center. Multiple challenges were identified that affected accrual with hMg often identified outside routine clinic encounters, physician reliance on IV Mg supplementation, and the difficultly of asking patients to complete on‐study tests without study personnel present (ie, follow up ECGs). In addition, ideally the definition of physician engagement would include all eligible patients that each physician could have approached for the study as the denominator. However, due to difficulties in obtaining pre‐treatment Mg level reports and confirming that physicians were able to review these results prior to treatment, we could not use this definition.

## 5 CONCLUSION

This study did not meet its feasibility endpoint due to the low number of patients accrued, which suggests that the integrated consent model is not optimal for randomization when the topic of interest occurs after initial management decisions. Oral Mg replacement strategies for patients who develop hMg from platinum and EGFRI based therapies are effective at preventing further Mg decline and are well tolerated. The optimal oral Mg supplement strategy is yet to be determined.

## CONFLICTS OF INTEREST

AA reports participating in the Novartis Canada Advisory Board on the use of Ribociclib, BH consults for Cornerstore Research, not related to this research project. All other authors have nothing to disclose.

## AUTHOR CONTRIBUTIONS

Conceptualization: M Vickers, M Clemons and D Fergusson.

Data Collection: A Awan, B Basulaiman, C Stober, M Clemons, J Hilton, W Al Ghareeb, R Goodwin, M Ibrahim, L Vandermeer, M Vickers

Formal Analysis: D Fergusson, R Mallick

Funding Acquisition: M. Vickers

Writing ‐ review and editing: all authors

Writing ‐original draft: A Awan, C Stober, M Clemons, M Vickers.

All authors have read and approved the final version of the manuscript.

Dr Michael Vickers had full access to all the data in this study and takes complete responsibility for the integrity of the data and the accuracy of the data analysis.

## TRANSPARENCY STATEMENT

Dr. Michael Vickers affirms that this manuscript is an honest, accurate, and transparent account of the study being reported; that no important aspects of the study have been omitted; and that any discrepancies from the study as planned and registered have been explained.

## Supporting information


**Data S1.** REaCT‐Mg consent script.

**Data S2.** CONSORT checklist.

## Data Availability

Due to the small number of participants, this study dataset contains potentially identifying and sensitive patient information. Access to data requests can be made to the Ottawa Health Science Network Research Ethics Board (613‐798‐5555 ext. 16 719 or rebadministration@toh.ca).
