# Supplementary material for: Oral magnesium supplements for cancer treatment‐induced hypomagnesemia: Results from a pilot randomized trial
Source: Health Sci Rep. 2021 Dec 14;4(4):e443. doi: 10.1002/hsr2.443 (PMC8669698; doi:10.1002/hsr2.443)
Supplement: Supplementary file 1 — Data S1. REaCT‐Mg consent script. [file HSR2-4-e443-s001.doc]

**Feasibility of using an Integrated Consent Model to Compare Two Standard of Care Regimens for the Management of Hypomagnesemia from Anti-Cancer therapies**

**REaCT-Mg OTT 15-03**

**Principal Investigator: Dr. M. Vickers, TOHCC**

“Our discussion today is a new approach of informing and consenting patients to participate in a study. The traditional approach is to provide a paper copy of the detailed information sheet and consent form for you to sign. Using this integrated model for consenting we will have a discussion and you may give a verbal consent to participate or not to participate. I will document our discussion and your decision in your progress notes that are part of your health records

As you are receiving Cisplatin, Carboplatin, Panitimumab or Cetuximab systemic therapy, there is a chance that you can develop low magnesium levels. Your recent bloodwork prior to today’s treatment has shown this to be the case. We therefore give patients medications to try to improve or stabilize the magnesium level. We can treat you with an oral supplement called magnesium oxide or an oral supplement called magnesium citrate.

They are both commonly used supplements to treat low magnesium levels and are started as tablets taken twice a day continuously. The doses and number of times a day you need to take these supplements may change depending on your magnesium levels in the future. They can both have side effects that are potentially mild and are significantly less than those that the chemotherapy causes. The most common side effect of both these supplements is diarrhea, which can be easily treated with anti-diarrhea medication. Because we really don't know if one is better than the other, some of the Oncologists at the Ottawa Hospital Cancer Centre involved in this study will randomly (like a flip of a coin, so that we can obtain an unbiased answer) assign participants one or the other supplement and then comparing the results over a period of 1 year. If you choose to participate there won't be any special procedures beyond the standard of care for patients with low magnesium. You will receive a prescription for the magnesium supplement that you will fill at the Ottawa Hospital Cancer Centre Pharmacy at no cost to you. You will receive a copy of this document for your reference.

If you choose to participate and then change your mind later, we can discuss together how to proceed.

Your participation in this study is voluntary. If you choose not to participate, your decision will not affect the care you receive at this Institution at this time, or in the future. You will not have any penalty or loss of benefits to which you are otherwise entitled to.

All research-related records will be kept for 10 years after termination of the study. No identifiable information will leave the Ottawa Hospital Cancer Centre. On all study related documentation, you will be identified with an independent study code rather than your name or other personal identifying information. The Ottawa Hospital Science Network Research Ethics Board (OHSN-REB) and the Ottawa Hospital Research Institute may review your original medical records and relevant study records for audit purposes, under the supervision of

Dr. M. Vickers.

If you have any questions about this study please, contact the Principal Investigator, Dr. M. Vickers at 613-737-7700 extension 70185.

The OHSN-REB has reviewed this protocol. The Board considers the ethical aspects of all research studies involving human participants at the Ottawa Hospital Cancer Centre. If you have any questions about your rights as a study participant, you may contact the Chairperson at 613-798-5555, extension 16719.

A description of this clinical trial will be available on [*http://www.ClinicalTrials.gov*](http://www.ClinicalTrials.gov/).  This website will not include information that can identify you. At most, the website will include a summary of the results. You can search this website at any time.

Do you have any questions?

You will receive a copy of this document for your reference.”
